# Supplementary material for: Influenza Vaccine Uptake in the United States before and during the COVID-19 Pandemic
Source: Vaccines (Basel). 2022 Sep 26;10(10):1610. doi: 10.3390/vaccines10101610 (PMC9612058; doi:10.3390/vaccines10101610)
Supplement: Supplementary file 1 [file vaccines-10-01610-s001.zip › vaccines-1905130-supplementary.pdf]

**Table S1. Demographic and clinical characteristics by influenza season, 2015-2016 through 2020-2021**

| Variable | 2015 - 2016 | 2016 - 2017 | 2017 - 2018 | 2018 - 2019 | 2019 - 2020 | 2020 - 2021 |
|---|---|---|---|---|---|---|
| | N=749,678 | N=2,408,739 | N=2,833,333 | N=2,869,709 | N=2,821,427 | N=2,365,991 |
| Age, y,[1] mean (SD) | 42.0 (21.7) | 43.0 (22.9) | 43.3 (23.0) | 44.5 (22.9) | 45.5 (22.9) | 47.2 (22.5) |
| Age, y,[1] median (IQR) | 46.0 (24 - 59) | 47.0 (22-61) | 47.0 (23-61) | 49.0 (25-62) | 49.0 (27-63) | 51.0 (30-64) |
| Age group, N (%) | | | | | | |
| 1- 4 y | 37,448 (5.0%) | 108,512 (4.5%) | 126,867 (4.5%) | 116,846 (4.1%) | 103,425 (3.7%) | 61,607 (2.6%) |
| 5 – 12 y | 66,328 (8.8%) | 237,059 (9.8%) | 273,181 (9.6%) | 251,007 (8.7%) | 232,718 (8.2%) | 158,506 (6.7%) |
| 13 – 17 y | 42,416 (5.7%) | 153,175 (6.4%) | 176,495 (6.2%) | 166,095 (5.8%) | 154,971 (5.5%) | 116,770 (4.9%) |
| 18 – 49 y | 268,982 (35.9%) | 793,880 (33.0%) | 932,223 (32.9%) | 944,608 (32.9%) | 921,787 (32.7%) | 800,813 (33.8%) |
| 50 – 64 y | 233,013 (31.1%) | 688,963 (28.6%) | 800,272 (28.2%) | 817,736 (28.5%) | 791,005 (28.0%) | 639,822 (27.0%) |
| 60 – 64 y | 76,052 (10.1%) | 234,231 (9.7%) | 279,960 (9.9%) | 293,695 (10.2%) | 286,066 (10.1%) | 227,419 (9.6%) |
| 65+ y | 101,491 (13.5%) | 427,150 (17.7%) | 524,295 (18.5%) | 573,417 (20.0%) | 617,521 (21.9%) | 588,473 (24.9%) |
| 65 – 69 y | 41,667 (5.6%) | 141,540 (5.9%) | 170,433 (6.0%) | 187,833 (6.5%) | 199,445 (7.1%) | 185,736 (7.9%) |
| 65 – 74 y | 66,888 (8.9%) | 252,859 (10.5%) | 309,198 (10.9%) | 336,736 (11.7%) | 360,440 (12.8%) | 341,661 (14.4%) |
| 75 – 84 y | 34,603 (4.6%) | 174,291 (7.2%) | 164,020 (5.8%) | 178,878 (6.2%) | 191,035 (6.8%) | 180,752 (7.6%) |
| 85+ y | 0 (0.0%) | 0 (0.0%) | 51,077 (1.8%) | 57,803 (2.0%) | 66,046 (2.3%) | 66,060 (2.8%) |
| Sex, N (%) | | | | | | |
| Female | 438,382 (58.5%) | 1,394,258 (57.9%) | 1,639,997 (57.9%) | 1,662,153 (57.9%) | 1,626,996 (57.7%) | 1,368,155 (57.8%) |
| Male | 311,296 (41.5%) | 1,014,481 (42.1%) | 1,193,336 (42.1%) | 1,207,556 (42.1%) | 1,194,431 (42.3%) | 997,836 (42.2%) |
| Race, N (%) | | | | | | |
| White | 488,588 (65.2%) | 1,492,642 (62.0%) | 1,668,553 (58.9%) | 1,630,393 (56.8%) | 1,550,464 (55.0%) | 1,287,333 (54.4%) |
| Asian | 19,579 (2.6%) | 70,176 (2.9%) | 89,267 (3.2%) | 93,707 (3.3%) | 93,513 (3.3%) | 70,929 (3.0%) |
| Black | 65,467 (8.7%) | 226,794 (9.4%) | 267,881 (9.5%) | 269,315 (9.4%) | 244,787 (8.7%) | 202,456 (8.6%) |

| | | | | | | |
|---|---|---|---|---|---|---|
| Other | 28,329 (3.8%) | 97,014 (4.0%) | 117,168 (4.1%) | 122,331 (4.3%) | 123,455 (4.4%) | 107,274 (4.5%) |
| Unknown/not Reported | 147,715 (19.7%) | 522,113 (21.7%) | 690,464 (24.4%) | 753,963 (26.3%) | 809,208 (28.7%) | 697,999 (29.5%) |
| Ethnicity, N (%) | | | | | | |
| Hispanic | 67,161 (9.0%) | 264,267 (11.0%) | 332,017 (11.7%) | 320,937 (11.2%) | 305,550 (10.8%) | 230,418 (9.7%) |
| Non-Hispanic | 611,815 (81.6%) | 1,960,516 (81.4%) | 2,313,873 (81.7%) | 2,350,394 (81.9%) | 2,315,708 (82.1%) | 1,966,464 (83.1%) |
| Unknown/not Reported | 70,702 (9.4%) | 183,956 (7.6%) | 187,443 (6.6%) | 198,378 (6.9%) | 200,169 (7.1%) | 169,109 (7.1%) |
| Baseline health status | | | | | | |
| High-risk patients, N (%) | 300,901 (40.1%) | 1,207,549 (50.1%) | 1,490,218 (52.6%) | 1,532,642 (53.4%) | 1,526,220 (54.1%) | 1,255,813 (53.1%) |
| Age 1 – 4 y | 7,883 (2.6%) | 32,948 (2.7%) | 39,393 (2.6%) | 36,222 (2.4%) | 32,755 (2.1%) | 17,359 (1.4%) |
| Age 5 – 12 y | 11,965 (4.0%) | 59,988 (5.0%) | 72,356 (4.9%) | 66,606 (4.3%) | 61,140 (4.0%) | 37,506 (3.0%) |
| Age 13-17 y | 6,698 (2.2%) | 35,166 (2.9%) | 43,598 (2.9%) | 40,483 (2.6%) | 37,787 (2.5%) | 26,084 (2.1%) |
| Age 18-49 y | 79,333 (26.4%) | 305,108 (25.3%) | 375,079 (25.2%) | 379,278 (24.7%) | 367,856 (24.1%) | 300,452 (23.9%) |
| Age 50-64 y | 121,027 (40.2%) | 425,142 (35.2%) | 520,072 (34.9%) | 531,979 (34.7%) | 512,201 (33.6%) | 395,674 (31.5%) |
| Age 65+ y | 73,995 (24.6%) | 349,197 (28.9%) | 439,720 (29.5%) | 478,074 (31.2%) | 514,481 (33.7%) | 478,738 (38.1%) |
| Non-high-risk patients N (%) | 448,777 (59.9%) | 1,201,190 (49.9%) | 1,343,115 (47.4%) | 1,337,067 (46.6%) | 1,295,207 (45.9%) | 1,110,178 (46.9%) |
| Age 1 – 4 y | 29,565 (6.6%) | 75,564 (6.3%) | 87,474 (6.5%) | 80,624 (6.0%) | 70,670 (5.5%) | 44,248 (4.0%) |
| Age 5 – 12 y | 54,363 (12.1%) | 177,071 (14.7%) | 200,825 (15.0%) | 184,401 (13.8%) | 171,578 (13.2%) | 121,000 (10.9%) |
| Age 13-17 y | 35,718 (8.0%) | 118,009 (9.8%) | 132,897 (9.9%) | 125,612 (9.4%) | 117,184 (9.0%) | 90,686 (8.2%) |
| Age 18-49 y | 189,649 (42.3%) | 488,772 (40.7%) | 557,144 (41.5%) | 565,330 (42.3%) | 553,931 (42.8%) | 500,361 (45.1%) |
| Age 50-64 y | 111,986 (25.0%) | 263,821 (22.0%) | 280,200 (20.9%) | 285,757 (21.4%) | 278,804 (21.5%) | 244,148 (22.0%) |
| Age 65+y | 27,496 (6.1%) | 77,953 (6.5%) | 84,575 (6.3%) | 95,343 (7.1%) | 103,040 (8.0%) | 109,735 (9.9%) |
| CCI (mean, SD) | 0.42 (1.04) | 0.74 (1.41) | 0.80 (1.49) | 0.83 (1.52) | 0.86 (1.57) | 0.86 (1.58) |
| CCI conditions, N (%) | | | | | | |
| Myocardial infarction | 6,889 (0.9%) | 39,528 (1.6%) | 49,426 (1.7%) | 52,370 (1.8%) | 52,991 (1.9%) | 44,963 (1.9%) |
| Congestive heart failure | 15,015 (2.0%) | 83,455 (3.5%) | 108,941 (3.8%) | 119,416 (4.2%) | 123,637 (4.4%) | 107,687 (4.6%) |
| Peripheral vascular disease | 18,049 (2.4%) | 118,244 (4.9%) | 155,663 (5.5%) | 171,158 (6.0%) | 186,129 (6.6%) | 160,757 (6.8%) |

| | | | | | | |
|---|---|---|---|---|---|---|
| Cerebrovascular disease | 16,444 (2.2%) | 88,710 (3.7%) | 109,488 (3.9%) | 117,072 (4.1%) | 123,599 (4.4%) | 104,119 (4.4%) |
| Dementia | 3,796 (0.5%) | 30,040 (1.2%) | 42,242 (1.5%) | 44,794 (1.6%) | 47,892 (1.7%) | 43,941 (1.9%) |
| Chronic pulmonary disease | 75,088 (10.0%) | 350,302 (14.5%) | 428,818 (15.1%) | 434,129 (15.1%) | 418,812 (14.8%) | 333,284 (14.1%) |
| Rheumatic disease | 13,076 (1.7%) | 56,234 (2.3%) | 69,445 (2.5%) | 72,991 (2.5%) | 76,105 (2.7%) | 64,311 (2.7%) |
| Peptic ulcer disease | 2,613 (0.3%) | 16,911 (0.7%) | 22,036 (0.8%) | 23,153 (0.8%) | 23,140 (0.8%) | 17,944 (0.8%) |
| Mild liver disease | 15,786 (2.1%) | 82,025 (3.4%) | 110,355 (3.9%) | 117,915 (4.1%) | 124,404 (4.4%) | 99,136 (4.2%) |
| Diabetes without chronic complications | 7,010 (0.9%) | 324,606 (13.5%) | 426,263 (15.0%) | 443,075 (15.4%) | 443,938 (15.7%) | 367,200 (15.5%) |
| Renal disease, mild to moderate | 19,447 (2.6%) | 89,948 (3.7%) | 109,928 (3.9%) | 121,369 (4.2%) | 129,914 (4.6%) | 113,956 (4.8%) |
| Diabetes with chronic complications | 20,324 (2.7%) | 111,581 (4.6%) | 163,138 (5.8%) | 180,420 (6.3%) | 189,518 (6.7%) | 159,307 (6.7%) |
| Hemiplegia or paraplegia | 2,953 (0.4%) | 16,173 (0.7%) | 20,763 (0.7%) | 21,311 (0.7%) | 20,700 (0.7%) | 17,054 (0.7%) |
| Any malignancy | 26,600 (3.5%) | 107,000 (4.4%) | 120,992 (4.3%) | 123,121 (4.3%) | 128,007 (4.5%) | 111,456 (4.7%) |
| Moderate to severe liver disease | 918 (0.1%) | 5,233 (0.2%) | 7,462 (0.3%) | 7,446 (0.3%) | 7,535 (0.3%) | 6,393 (0.3%) |
| Severe renal disease | 3,857 (0.5%) | 21,079 (0.9%) | 26,636 (0.9%) | 25,961 (0.9%) | 26,346 (0.9%) | 21,976 (0.9%) |
| HIV infection, no AIDS | 2,143 (0.3%) | 7,619 (0.3%) | 9,392 (0.3%) | 9,953 (0.3%) | 10,218 (0.4%) | 8,352 (0.4%) |
| Metastatic solid tumor | 2,083 (0.3%) | 10,964 (0.5%) | 14,938 (0.5%) | 15,045 (0.5%) | 16,081 (0.6%) | 14,779 (0.6%) |
| AIDS | 290 (0.0%) | 1,186 (0.0%) | 1,440 (0.1%) | 1,475 (0.1%) | 1,476 (0.1%) | 1,182 (0.0%) |
| Baseline cardiovascular and other risk | | | | | | |
| Asthma and chronic obstructive pulmonary disease, N (%) | 54,952 (7.3%) | 303,553 (12.6%) | 377,200 (13.3%) | 383,344 (13.4%) | 371,985 (13.2%) | 297,619 (12.6%) |
| Myocardial infarction-related IP admission, N (%) | 1,452 (0.2%) | 8,113 (0.3%) | 9,919 (0.4%) | 10,881 (0.4%) | 11,388 (0.4%) | 9,838 (0.4%) |
| Ischemic stroke-related IP admission, N (%) | 1,148 (0.2%) | 6,987 (0.3%) | 8,794 (0.3%) | 9,929 (0.3%) | 10,372 (0.4%) | 8,924 (0.4%) |
| Transient ischemic attack–related IP admission, N (%) | 524 (0.1%) | 3,357 (0.1%) | 4,211 (0.1%) | 4,120 (0.1%) | 4,188 (0.1%) | 3,210 (0.1%) |
| Hypercholesteremia, N (%) | 42,741 (5.7%) | 56,952 (2.4%) | 202,499 (7.1%) | 230,124 (8.0%) | 229,622 (8.1%) | 182,592 (7.7%) |
| Hypertension, N (%) | 179,695 (24.0%) | 746,936 (31.0%) | 908,727 (32.1%) | 940,752 (32.8%) | 940,593 (33.3%) | 791,933 (33.5%) |

| | | | | | | |
|---|---|---|---|---|---|---|
| Type 2 diabetes, N (%) | 81,826 (10.9%) | 335,215 (13.9%) | 427,850 (15.1%) | 445,031 (15.5%) | 447,490 (15.9%) | 370,770 (15.7%) |
| BMI (mean, SD) | 27.9 (7.6) | 27.9 (7.6) | 28.1 (7.6) | 28.3 (7.6) | 28.4 (7.5) | 28.7 (7.4) |
| BMI, median (IQR) | 27.3 (22.7 - 32.6) | 27.2(22.7 - 32.6) | 27.3(22.9-32.7) | 27.6 (23.0-33.0) | 27.8 (23.3-33.0) | 28.0 (23.9-33.0) |
| Smoking status, N (%) | | | | | | |
| Current | 21,906 (2.9%) | 78,732 (3.3%) | 93,402 (3.3%) | 102,012 (3.6%) | 96,226 (3.4%) | 86,909 (3.7%) |
| Former | 16,482 (2.2%) | 50,317 (2.1%) | 59,756 (2.1%) | 64,785 (2.3%) | 64,025 (2.3%) | 57,210 (2.4%) |
| Never | 63,159 (8.4%) | 169,168 (7.0%) | 189,577 (6.7%) | 205,235 (7.2%) | 124,431 (4.4%) | 78,950 (3.3%) |
| Unknown | 648,131 (86.5%) | 2,110,522 (87.6%) | 2,490,598 (87.9%) | 2,497,677 (87.0%) | 2,536,745 (89.9%) | 2,142,922 (90.6%) |
| Healthcare Resource Utilization in Baseline | | | | | | |
| Patients with OP visit, N (%) | 680,765 (90.8%) | 2,246,121 (93.2%) | 2,644,062 (93.3%) | 2,677,805 (93.3%) | 2,624,471 (93.0%) | 2,161,466 (91.4%) |
| Number of all-cause OP visits (mean, SD) | 5.1 (6.2) | 6.7 (8.4) | 7.0 (9.3) | 7.1 (9.6) | 7.2 (9.5) | 6.6 (8.9) |
| Patients with ED visit, N (%) | 112,350 (15.0%) | 579,609 (24.1%) | 705,694 (24.9%) | 712,811 (24.8%) | 682,029 (24.2%) | 516,426 (21.8%) |
| Number of all-cause ED visits (mean, SD) | 0.31 (1.5) | 0.60 (2.5) | 0.63 (2.6) | 0.64 (2.9) | 0.62 (3.0) | 0.55 (2.9) |
| Patients with IP admission, N (%) | 40,405 (5.4%) | 225,191 (9.3%) | 279,881 (9.9%) | 299,954 (10.5%) | 295,717 (10.5%) | 243,271 (10.3%) |
| Number of all-cause IP admissions (mean, SD) | 0.08 (0.5) | 0.16 (0.7) | 0.20 (1.1) | 0.21 (1.2) | 0.22 (1.2) | 0.20 (1.1) |
| Type of vaccine, N (%) | 231,561 (30.9%) | 757,141 (31.4%) | 918,449 (32.4%) | 970,332 (33.8%) | 953,090 (33.8%) | 838,286 (35.4%) |
| Adjuvanted | 8 (0.0%) | 1,921 (0.1%) | 16,522 (0.6%) | 32,996 (1.1%) | 49,014 (1.7%) | 71,714 (3.0%) |
| LAIV | 13,094 (1.7%) | 140 (0.0%) | 95 (0.0%) | 4,036 (0.1%) | 3,047 (0.1%) | 6,474 (0.3%) |
| Cell-based | 2,007 (0.3%) | 9,832 (0.4%) | 78,223 (2.8%) | 103,847 (3.6%) | 132,510 (4.7%) | 115,388 (4.9%) |
| Recombinant | 127 (0.0%) | 390 (0.0%) | 1,570 (0.1%) | 26,475 (0.9%) | 51,834 (1.8%) | 60,165 (2.5%) |
| High dose | 23,061 (3.1%) | 116,567 (4.8%) | 147,664 (5.2%) | 157,814 (5.5%) | 136,820 (4.8%) | 127,433 (5.4%) |
| Egg-based | 193,264 (25.8%) | 628,291 (26.1%) | 674,375 (23.8%) | 645,164 (22.5%) | 579,865 (20.6%) | 457,112 (19.3%) |
| Place of Vaccination, N (%) | | | | | | |
| Office/other outpatient | 191,389 (25.5%) | 630,263 (26.2%) | 725,516 (25.6%) | 737,537 (25.7%) | 674,742 (23.9%) | 532,457 (22.5%) |
| Pharmacy | 38,827 (5.2%) | 119,249 (5.0%) | 184,342 (6.5%) | 223,800 (7.8%) | 269,907 (9.6%) | 301,516 (12.7%) |

| | | | | | | |
|---|---|---|---|---|---|---|
| Hospital | 829 (0.1%) | 4,766 (0.2%) | 5,083 (0.2%) | 5,033 (0.2%) | 4,396 (0.2%) | 1,656 (0.1%) |
| Other | 516 (0.1%) | 2,863 (0.1%) | 3,508 (0.1% | 3,962 (0.1%) | 4,045 (0.1%) | 2,657 (0.1%) |
| Timing of Vaccination, N (%) | | | | | | |
| August | 3,917 (0.5%) | 20,657 (0.9%) | 35,930 (1.3%) | 23,578 (0.8%) | 22,833 (0.8%) | 43,484 (1.8%) |
| September | 42,705 (5.7%) | 171,074 (7.1%) | 204,576 (7.2%) | 207,994 (7.2%) | 199,625 (7.1%) | 270,133 (11.4%) |
| October | 102,041 (13.6%) | 295,472 (12.3%) | 353,243 (12.5%) | 437,049 (15.2%) | 411,033 (14.6%) | 341,406 (14.4%) |
| November | 49,844 (6.6%) | 157,209 (6.5%) | 174,932 (6.2%) | 183,493 (6.4%) | 183,753 (6.5%) | 120,571 (5.1%) |
| December | 22,734 (3.0%) | 71,670 (3.0%) | 79,226 (2.8%) | 70,884 (2.5%) | 83,380 (3.0%) | 44,856 (1.9%) |
| January | 10,320 (1.4%) | 41,059 (1.7%) | 70,542 (2.5%) | 47,334 (1.6%) | 52,466 (1.9%) | 17,836 (0.8%) |
| Vaccinated, N (%) August to May [2] | 238,869 (31.9%) | 782,502 (32.5%) | 957,087 (33.8%) | 998,648 (34.8%) | 988,774 (35.0%) | 846,988 (35.8%) |
| Vaccination between February and May | 1.1% | 1.2% | 1.6% | 1.2% | 1.4% | 0.4% |
| February, N (%) | 4,973 (0.7%) | 18,067 (0.8%) | 32,057 (1.1%) | 19,159 (0.7%) | 23,832 (0.8%) | 6,549 (0.3%) |
| March, N (%) | 2,425 (0.3%) | 7,595 (0.3%) | 8,231 (0.3%) | 9,479 (0.3%) | 14,243 (0.5%) | 2,857 (0.1%) |
| April, N (%) | 662 (0.1%) | 2,237 (0.1%) | 2,580 (0.1%) | 3,231 (0.1%) | 1,453 (0.1%) | 865 (0.0%) |
| May, N (%) | 327 (0.0%) | 1,116 (0.0%) | 1,129 (0.0%) | 1,339 (0.0%) | 998 (0.0%) | 254 (0.0%) |

BMI: body mass index; CCI: Charlson Comorbidity Index; ED: emergency department; IP: inpatient; IQR: interquartile range; LAIV: live attenuated influenza virus; OP: outpatient; SD: standard deviation

[1]Age as reported at the start of the influenza season.

[2]The sum of counts of those vaccinated August-May will be equal to or higher than the total number in this row. Per patient selection, patients were required to have only 1 vaccination (or 2 if age < 9 years) between August and January but may have had a second (or third) vaccination record between February and May and therefore may have been double counted in the February to May time period.

**Table S2. Current Procedural Terminology, Vaccine Administered, and National Drug Codes**

| Vaccine Type | Code Type | Codes |
|---|---|---|
| Adjuvanted | CPT | 90653, 90689 |
| | CVX | 168 |
| | NDC | 66521000001, 66521000011, 70461000101, 70461000111, 70461000201, 70461000211, 70461001803, 70461001804, 70461001903, 70461001904 |
| Cell-based | CPT | 90674, 90756 |
| | CVX | 171, 186 |
| | NDC | 62577061401, 62577061411, 70461020001, 70461020011, 70461020101, 70461020111, 70461030110, 70461030112, 70461031803, 70461031804, 70461041810, 70461041811, 70461031903, 70461031904, 70461041911, 70461041910 |
| High Dose | CPT | 90662 |
| | CVX | 135, 197 |
| | NDC | 49281039765, 49281039788, 49281039965, 49281039988, 49281040165, 49281040188, 49281040365, 49281040388, 49281040588, 49281040565 |
| LAIV | CPT | 90672, 90660 |
| | CVX | 111, 149 |
| | NDC | 66019030610, 66019030601, 66019030510, 66019030501, 66019030410, 66019030401, 66019030310, 66019030301, 66019030210, 66019030201 |
| Recombinant | CPT | 90673, 90682 |
| | CVX | 155, 185 |
| | NDC | 42874001501, 42874001510, 42874001601, 42874001610, 42874001701, 42874001710, 42874011701, 42874011710, 49281071810, 49281071888, 49281071910, 49281071988 |
| TIVe | CPT | 90654, 90655, 90656, 90657, 90658 |
| | CVX | 140, 141, 168 |
| | NDC | 33332011510, 33332011511, 33332001501, 33332001502, 33332011611, 33332011610, 33332001601, 33332001602, 33332001701, 33332001702, 33332011710, 33332011711, 33332001801, 33332001802, 33332011810, 33332011811, 66521011802, 66521011810, 66521011811, 66521011812, 70461011902, 70461011910, 70461011911, 70461011912, 70461012002, 70461012012, 70461012010, 70461012011 |
| QIVe | CPT | 90630, 90685, 90686, 90687, 90688 |
| | CVX | 150, 158, 161, 166 |

| Vaccine Type | Code Type | Codes |
|---|---|---|
| | NDC | 19515089801, 19515089811, 19515089441, 19515089452, 19515090301, 19515090311, 19515090841, 19515090852, 19515089601, 19515089611, 19515091241, 19515091252, 19515090001, 19515090011, 19515090941, 19515090952, 19515089701, 19515089711, 19515090641, 19515090652, 33332031601, 33332031602, 33332031701, 33332031702, 33332041710, 33332041711, 33332031801, 33332031802, 33332041810, 33332041811, 33332021920, 33332021921, 33332031901, 33332031902, 33332041910, 33332041911, 49281041510, 49281041550, 49281041558, 49281041588, 49281051500, 49281051525, 49281062315, 49281062378, 49281041610, 49281041650, 49281041658, 49281041688, 49281051600, 49281051625, 49281062515, 49281062578, 49281071040, 49281071048, 49281041710, 49281041750, 49281041758, 49281041788, 49281051700, 49281051725, 49281062715, 49281062778, 49281071240, 49281071248, 49281041800, 49281041810, 49281041850, 49281041858, 49281041888, 49281051800, 49281051825, 49281062915, 49281062978, 49281041910, 49281041950, 49281041958, 49281041988, 49281051900, 49281051925, 49281063115, 49281063178, 58160090341, 58160090352, 58160090541, 58160090552, 58160090741, 58160090752, 58160089841, 58160089852, 58160089641, 58160089652 |

CPT = Current Procedural Terminology; CVX = Vaccine Administered codes; LAIV = Live attenuated influenza vaccine; NDC = National Drug Code; QIVe = Quadrivalent influenza vaccine; TIVe = Trivalent influenza vaccine

**Table S3. Medical conditions that increase risk of experiencing serious influenza complications [1]**

| |
|---|
| • Asthma<br>• Neurologic and neurodevelopmental conditions<br>• Blood disorders (e.g., sickle cell disease)<br>• Chronic lung disease (e.g., COPD, cystic fibrosis)<br>• Endocrine disorders (e.g., diabetes mellitus)<br>• Heart disease (e.g., congenital heart disease, congestive heart failure, coronary artery disease)<br>• Kidney diseases<br>• Liver disorders<br>• Metabolic disorders (e.g., inherited metabolic disorders, mitochondrial disorders)<br>• Obesity (BMI of 40 or higher)<br>• Weakened immune system due to disease (e.g., HIV or AIDS, leukemia) or medications (e.g., chemotherapy or radiation treatment for cancer, chronic corticosteroids)<br>• Stroke |

BMI: body mass index; COPD: chronic obstructive pulmonary disease.

Note: Additional factors that are known to be associated with a higher risk of serious complications from an influenza infection that have not been included in the high-risk criteria, include age (over 65), people living in nursing homes, and people from certain racial and ethnic minority backgrounds.

**Table S4. Vaccination history cohorts**

| | Vaccination Season | | | | |
|---|---|---|---|---|---|
| **Influenza Vaccination History Cohort** | **2015-2016** | **2016-2017** | **2017-2018** | **2018-2019** | **2019-2020** |
| Consistently Vaccinated | X | X | X | X | X |
| Previous Adopters (Example 1) | | | | X | X |
| Previous Adopters (Example 2) | | | X | X | X |
| Previous Adopters (Example 3) | | X | X | X | X |
| Vaccinated 2019-2020 Only | | | | | X |
| Not Vaccinated | | | | | |
| Inconsistently Vaccinated | X | | X | | X |

**Table S5. Cohort selection for determining influenza vaccine uptake patterns, 2016-2017 through 2020-2021**

| Selection Criterion | 2015 - 2016 | | 2016 - 2017 | | 2017 - 2018 | | 2018 - 2019 | | 2019 - 2020 | | 2020 - 2021 | |
|---|---|---|---|---|---|---|---|---|---|---|---|---|
| | N | % | N | % | N | % | N | % | N | % | N | % |
| 1) Patient has EMR activity in the Veradigm® EMR during the influenza vaccination season | 33,332,60 2 | | 30,400,114 | | 29,491,859 | | 26,605,530 | | 25,469,759 | | 24,760,625 | |
| 2) Patient has EMR activity in the Veradigm® EMR more than 12 months before the start of the influenza vaccination season | 18,095,10 9 | 54.3% | 19,942,921 | 65.6% | 20,478,643 | 69.4% | 19,220,773 | 72.2% | 18,867,531 | 74.1% | 18,436,795 | 74.5% |
| 3) Patient is continuously enrolled in the Linked Dataset from 12 months before the start of the flu vaccination season through the duration of the influenza vaccination season | 1,021,012 | 5.6% | 3,167,010 | 15.9% | 3,632,847 | 17.7% | 3,577,172 | 18.6% | 3,406,207 | 18.1% | 2,758,041 | 15.0% |
| 4) Patients aged 1+ at the start of the influenza season | 1,020,911 | 100.0% | 3,166,760 | 100.0% | 3,632,558 | 100.0% | 3,576,866 | 100.0% | 3,405,930 | 100.0% | 2,757,835 | 100.0% |
| 5) Patient does not have missing data in the covariate fields age, gender, or geographic region | 767,423 | 75.2% | 2,468,027 | 77.9% | 2,916,523 | 80.3% | 2,963,695 | 82.9% | 2,913,532 | 85.5% | 2,445,495 | 88.7% |
| 6) Patients aged 9+ do not have >1 influenza vaccine recorded; patients under 9 do not have >2 influenza vaccines recorded | 749,678 | 97.7% | 2,408,739 | 97.6% | 2,833,333 | 97.1% | 2,869,709 | 96.8% | 2,821,427 | 96.8% | 2,365,991 | 96.7% |

EMR: Electronic medical record

## Table S6. Cohort selection to determine characteristics of recipients of the 2020-2021 season influenza vaccine compared with nonrecipients

| Selection Criterion | 2015 - 2021 |
|---|---|
| | N (%) |
| 1) Closed claims data were available for continuously enrolled patients in the Linked Dataset from the start of the baseline period for the 2020–2021 season through the end of that season | 9,519,492 |
| 2) Patient is continuously enrolled in the closed claims data from the start through the end of all the influenza vaccination seasons analyzed (August 1, 2015, through January 31, 2021) | 4,369,665 (45.90%) |
| 3) Patient has activity in the EMR data once during each influenza season analyzed | 403,728 (9.24%) |
| 4) Patient has activity in the EMR data at least 12 months before the start of the 2020 – 2021 influenza vaccination season | 403,728 (100.00%) |
| 5) Patient is at least aged 5+ at the start of the 2020-2021 influenza season | 403,657 (99.98%) |
| 6) Patient does not have missing data in the covariate fields age, gender, or geographic region | 403,393 (99.93%) |
| 7) Patients aged 9+ do not have >1 influenza vaccine recorded in any season; patients under 9 do not have >2 influenza vaccines recorded | 325,389 (80.66%) |
| Received a vaccine in the 2020-2021influenza season | 138,824 (42.7%) |
| Consistently vaccinated | 36,123 (26.0%) |
| Not vaccinated | 16,376 (11.8%) |
| Vaccinated in the 2019-2020 influenza season only | 7,601 (5.5%) |
| Previous adopters | 58,649 (42.2%) |
| Inconsistently vaccinated | 20,075 (14.5%) |
| Did not receive a vaccine in the 2020-2021influenza season | 186,565 (57.3%) |
| Consistently vaccinated | 7,017 (3.8%) |
| Not vaccinated | 99,105 (53.1%) |
| Vaccinated in the 2019-2020 influenza season only | 7,336 (3.9%) |
| Previous adopters | 38,553 (20.7%) |
| Inconsistently vaccinated | 34,554 (18.5%) |

EMR = electronic medical record

**Table S7. Characteristics of individuals continuously enrolled in the Linked Dataset from the 2015-2016 through the 2020-2021 influenza seasons, based on vaccination status of the 2020-2021 season**

| Variable | Vaccinated 2020 - 2021 | Unvaccinated 2020 - 20201 |
|---|---|---|
| | N=138,824 | N=186,565 |
| **Age in y,[1] mean (SD)** | 55.0 (22.9) | 54.3 (20.5) |
| **Age in y,[1] median (IQR)** | 61 (45-72) | 58 (44-69) |
| **Age group, N (%)** | | |
| 5 – 12 y | 12,797 (9.2%) | 9,480 (5.1%) |
| 13 – 17 y | 6,771 (4.9%) | 6,884 (3.7%) |
| 18 – 49 y | 21,162 (15.2%) | 45,359 (24.3%) |
| 50 – 64 y | 43,977 (31.7%) | 63,782 (34.2%) |
| 60 – 64 y | 18,961 (13.7%) | 23,367 (12.5%) |
| 65+ y | 54,117 (39.0%) | 61,060 (32.7%) |
| 65 – 69 y | 13,075 (9.4%) | 15,971 (8.6%) |
| 65 – 74 y | 27,511 (19.8%) | 31,910 (17.1%) |
| 75 – 84 y | 19,418 (14.0%) | 20,681 (11.1%) |
| 85+ y | 7,188 (5.2%) | 8,469 (4.5%) |
| **Sex, N (%)** | | |
| Female | 79,433 (57.2%) | 109,072 (58.5%) |

| | | |
|---|---|---|
| Male | 59,391 (42.8%) | 77,493 (41.5%) |
| **Race, N (%)** | | |
| White | 97,085 (69.9%) | 120,835 (64.8%) |
| Asian | 5,533 (4.0%) | 4,874 (2.6%) |
| Black | 9,776 (7.0%) | 19,801 (10.6%) |
| Other | 7,050 (5.1%) | 10,346 (5.5%) |
| Unknown/not reported | 19,380 (14.0%) | 30,709 (16.5%) |
| **Ethnicity, N (%)** | | |
| Hispanic | 12,725 (9.2%) | 18,983 (10.2%) |
| Non-Hispanic | 118,038 (85.0%) | 159,275 (85.4%) |
| Unknown/not reported | 8,061 (5.8%) | 8,307 (4.5%) |
| **Baseline health status** | | |
| High-risk patients, N (%) | 94,227 (67.9%) | 117,990 (63.2%) |
| Non-high-risk patients, N (%) | 44,597 (32.1%) | 68,575 (36.8%) |
| CCI (mean, SD) | 1.20 (1.77) | 1.09 (1.71) |
| CCI conditions (N, %) | | |
| Myocardial infarction | 3,558 (2.6%) | 4,294 (2.3%) |
| Congestive heart failure | 9,064 (6.5%) | 11,391 (6.1%) |
| Peripheral vascular disease | 13,972 (10.1%) | 16,564 (8.9%) |
| Cerebrovascular disease | 8,619 (6.2%) | 10,431 (5.6%) |
| Dementia | 2,821 (2.0%) | 4,207 (2.3%) |
| Chronic pulmonary disease | 24,325 (17.5%) | 29,615 (15.9%) |

| | | |
|---|---|---|
| Rheumatic disease | 5,240 (3.8%) | 6,716 (3.6%) |
| Peptic ulcer disease | 1,214 (0.9%) | 1,471 (0.8%) |
| Mild liver disease | 6,975 (5.0%) | 8,518 (4.6%) |
| Diabetes without chronic complications | 30,952 (22.3%) | 38,369 (20.6%) |
| Renal disease, mild to moderate | 10,329 (7.4%) | 11,978 (6.4%) |
| Diabetes with chronic complications | 14,576 (10.5%) | 16,407 (8.8%) |
| Hemiplegia or paraplegia | 1,126 (0.8%) | 1,687 (0.9%) |
| Any malignancy | 10,280 (7.4%) | 11,741 (6.3%) |
| Moderate to severe liver disease | 402 (0.3%) | 530 (0.3%) |
| Severe renal disease | 1,615 (1.2%) | 2,169 (1.2%) |
| HIV infection, no AIDS | 563 (0.4%) | 768 (0.4%) |
| Metastatic solid tumor | 1,116 (0.8%) | 1,469 (0.8%) |
| AIDS | 67 (0.0%) | 84 (0.0%) |
| Baseline cardiovascular and other risk | | |
| Asthma and chronic obstructive pulmonary disease, N (%) | 21,939 (15.8%) | 26,426 (14.2%) |
| Myocardial infarction–related IP admission, N (%) | 679 (0.5%) | 913 (0.5%) |
| Ischemic stroke–related IP admission, N (%) | 506 (0.4%) | 775 (0.4%) |
| Transient ischemic attack–related IP admission, N (%) | 205 (0.1%) | 287 (0.2%) |
| Hypercholesteremia, N (%) | 16,658 (12.0%) | 17,661 (9.5%) |
| Hypertension, N (%) | 66,348 (47.8%) | 80,593 (43.2%) |
| Type 2 diabetes, N (%) | 31,411 (22.6%) | 38,874 (20.8%) |

| | | |
|---|---|---|
| BMI, mean (SD) | 28.8 (7.2) | 29.5 (7.2) |
| BMI, median (IQR) | 28 (24.0 - 33.0) | 28.74 (24.6 - 33.8) |
| Smoking status, N (%) | | |
| Current | 4,763 (3.4%) | 7,676 (4.1%) |
| Former | 4,238 (3.1%) | 5,117 (2.7%) |
| Never | 5,732 (4.1%) | 6,730 (3.6%) |
| Unknown | 124,091 (89.4%) | 167,042 (89.5%) |
| **Healthcare Resource Utilization in Baseline** | | |
| Patients with OP visit, N (%) | 136,848 (98.6%) | 179,867 (96.4%) |
| Number of all-cause OP visits, mean (SD) | 8.3 (10.0) | 7.3 (9.1) |
| Patients with ED visit, N (%) | 25,575 (18.4%) | 38,458 (20.6%) |
| Number of all-cause ED visits, mean (SD) | 0.49 (3.8) | 0.52 (3.2) |
| Patients with IP admission, N (%) | 13,608 (9.8%) | 19,606 (10.5%) |
| Number of all-cause IP admissions, mean (SD) | 0.17 (0.87) | 0.21 (1.10) |

[1]Age as of 2020

BMI: body mass index; CCI: Charlson Comorbidity Index; ED: emergency department; IP: inpatient; IQR: interquartile range; OP: outpatient; SD: standard deviation

**Table S8. Demographic and clinical characteristics by influenza vaccination history during the influenza seasons from 2015-2016 through 2019-2020**

| Variable | Consistently vaccinated | Previous adopters | Inconsistently vaccinated | Vaccinated 2019- 2020 only | Not vaccinated |
|---|---|---|---|---|---|
| | N=43,140 | N=97,202 | N=54,629 | N=14,937 | N=115,481 |
| **Age in y,[1] mean (SD)** | 54.0 (26.8) | 56.2 (22.7) | 53.6 (21.1) | 54.8 (19.2) | 54.0 (18.7) |
| **Age in y,[1] median (IQR)** | 63 (29 - 75) | 61 (46 - 73) | 58 (43 - 68) | 58 (45 - 67) | 57 (45 - 66) |
| **Age group, N (%)** | | | | | |
| 5 – 12 y | 6,263 (14.5%) | 7,839 (8.1%) | 3,358 (6.1%) | 614 (4.1%) | 4,203 (3.6%) |
| 13 – 17 y | 3,181 (7.4%) | 4,146 (4.3%) | 2,215 (4.1%) | 473 (3.2%) | 3,640 (3.2%) |
| 18 – 49 y | 4,223 (9.8%) | 15,332 (15.8%) | 12,904 (23.6%) | 3,571 (23.9%) | 30,491 (26.4%) |
| 50 – 64 y | 9,668 (22.4%) | 29,349 (30.2%) | 18,448 (33.8%) | 5,517 (36.9%) | 44,777 (38.8%) |
| 60 – 64 y | 4,570 (10.6%) | 12,472 (12.8%) | 7,053 (12.9%) | 2,170 (14.5%) | 16,063 (13.9%) |
| 65+ y | 19,805 (45.9%) | 40,536 (41.7%) | 17,704 (32.4%) | 4,762 (31.9%) | 32,370 (28.0%) |
| 65 – 69 y | 3,616 (8.4%) | 9,467 (9.7%) | 4,914 (9.0%) | 1,513 (10.1%) | 9,536 (8.3%) |
| 65 – 74 y | 8,579 (19.9%) | 20,156 (20.7%) | 9,633 (17.6%) | 2,772 (18.6%) | 18,281 (15.8%) |
| 75 – 84 y | 7,974 (18.5%) | 14,384 (14.8%) | 5,770 (10.6%) | 1,496 (10.0%) | 10,475 (9.1%) |
| 85+ y | 3,252 (7.5%) | 5,996 (6.2%) | 2,301 (4.2%) | 494 (3.3%) | 3,614 (3.1%) |
| **Sex, N (%)** | | | | | |
| Female | 24,458 (56.7%) | 55,455 (57.1%) | 31,603 (57.9%) | 8,705 (58.3%) | 68,284 (59.1%) |
| Male | 18,682 (43.3%) | 41,747 (42.9%) | 23,026 (42.1%) | 6,232 (41.7%) | 47,197 (40.9%) |
| **Race, N (%)** | | | | | |
| White | 32,313 (74.9%) | 67,177 (69.1%) | 36,048 (66.0%) | 9,943 (66.6%) | 72,439 (62.7%) |
| Asian | 1,841 (4.3%) | 3,528 (3.6%) | 1,766 (3.2%) | 465 (3.1%) | 2,807 (2.4%) |
| Black | 2,216 (5.1%) | 7,813 (8.0%) | 5,540 (10.1%) | 1,242 (8.3%) | 12,766 (11.1%) |
| Other | 1,669 (3.9%) | 5,192 (5.3%) | 3,279 (6.0%) | 868 (5.8%) | 6,388 (5.5%) |

| | | | | | |
|---|---|---|---|---|---|
| Unknown/not reported | 5,101 (11.8%) | 13,492 (13.9%) | 7,996 (14.6%) | 2,419 (16.2%) | 21,081 (18.3%) |
| **Ethnicity, N (%)** | | | | | |
| Hispanic | 3,232 (7.5%) | 8,998 (9.3%) | 5,851 (10.7%) | 1,521 (10.2%) | 12,106 (10.5%) |
| Non-Hispanic | 37,288 (86.4%) | 83,182 (85.6%) | 46,148 (84.5%) | 12,674 (84.8%) | 98,021 (84.9%) |
| Unknown/not reported | 2,620 (6.1%) | 5,022 (5.2%) | 2,630 (4.8%) | 742 (5.0%) | 5,354 (4.6%) |
| **Baseline health status** | | | | | |
| High-risk patients, N (%) | 29,108 (67.5%) | 67,909 (69.9%) | 35,283 (64.6%) | 10,023 (67.1%) | 69,894 (60.5%) |
| Non-high-risk patients, N (%) | 14,032 (32.5%) | 29,293 (30.1%) | 19,346 (35.4%) | 4,914 (32.9%) | 45,587 (39.5%) |
| CCI (Mean, SD) | 1.2 (1.79) | 1.3 (1.87) | 1.1 (1.75) | 1.1 (1.72) | 1.0 (1.58) |
| CCI conditions, N (%) | | | | | |
| Myocardial infarction | 1,070 (2.5%) | 2,825 (2.9%) | 1,273 (2.3%) | 400 (2.7%) | 2,284 (2.0%) |
| Congestive heart failure | 2,952 (6.8%) | 7,380 (7.6%) | 3,346 (6.1%) | 937 (6.3%) | 5,840 (5.1%) |
| Peripheral vascular disease | 4,841 (11.2%) | 10,625 (10.9%) | 4,762 (8.7%) | 1,329 (8.9%) | 8,979 (7.8%) |
| Cerebrovascular disease | 2,779 (6.4%) | 6,815 (7.0%) | 3,062 (5.6%) | 833 (5.6%) | 5,561 (4.8%) |
| Dementia | 973 (2.3%) | 2,690 (2.8%) | 1,250 (2.3%) | 293 (2.0%) | 1,822 (1.6%) |
| Chronic pulmonary disease | 7,974 (18.5%) | 18,184 (18.7%) | 9,446 (17.3%) | 2,446 (16.4%) | 15,890 (13.8%) |
| Rheumatic disease | 1,542 (3.6%) | 3,920 (4.0%) | 2,134 (3.9%) | 607 (4.1%) | 3,753 (3.2%) |
| Peptic ulcer disease | 348 (0.8%) | 908 (0.9%) | 469 (0.9%) | 135 (0.9%) | 825 (0.7%) |
| Mild liver disease | 1,941 (4.5%) | 5,026 (5.2%) | 2,588 (4.7%) | 820 (5.5%) | 5,118 (4.4%) |
| Diabetes without chronic complications | 9,270 (21.5%) | 23,172 (23.8%) | 11,846 (21.7%) | 3,179 (21.3%) | 21,854 (18.9%) |
| Renal disease, mild to moderate | 3,432 (8.0%) | 8,140 (8.4%) | 3,679 (6.7%) | 974 (6.5%) | 6,082 (5.3%) |
| Diabetes with chronic complications | 4,822 (11.2%) | 10,981 (11.3%) | 5,127 (9.4%) | 1,360 (9.1%) | 8,693 (7.5%) |
| Hemiplegia or paraplegia | 368 (0.9%) | 905 (0.9%) | 507 (0.9%) | 145 (1.0%) | 888 (0.8%) |
| Any malignancy | 3,356 (7.8%) | 7,695 (7.9%) | 3,514 (6.4%) | 1,023 (6.8%) | 6,433 (5.6%) |
| Moderate to severe liver disease | 134 (0.3%) | 336 (0.3%) | 148 (0.3%) | 57 (0.4%) | 257 (0.2%) |

| | | | | | |
|---|---|---|---|---|---|
| Severe renal disease | 378 (0.9%) | 1,392 (1.4%) | 707 (1.3%) | 194 (1.3%) | 1,113 (1.0%) |
| HIV infection, no AIDS | 141 (0.3%) | 480 (0.5%) | 296 (0.5%) | 64 (0.4%) | 350 (0.3%) |
| Metastatic solid tumor | 365 (0.8%) | 907 (0.9%) | 431 (0.8%) | 94 (0.6%) | 788 (0.7%) |
| AIDS | 20 (0.0%) | 60 (0.1%) | 26 (0.0%) | 10 (0.1%) | 35 (0.0%) |
| Baseline cardiovascular and other risk | | | | | |
| Asthma and chronic obstructive pulmonary disease, N (%) | 7,279 (16.9%) | 16,528 (17.0%) | 8,420 (15.4%) | 2,181 (14.6%) | 13,957 (12.1%) |
| Myocardial infarction–related IP admission, N (%) | 201 (0.5%) | 569 (0.6%) | 265 (0.5%) | 73 (0.5%) | 484 (0.4%) |
| Ischemic stroke–related IP admission, N (%) | 167 (0.4%) | 442 (0.5%) | 224 (0.4%) | 57 (0.4%) | 391 (0.3%) |
| Transient ischemic attack–related IP admission, N (%) | 63 (0.1%) | 169 (0.2%) | 95 (0.2%) | 25 (0.2%) | 140 (0.1%) |
| Hypercholesteremia, N (%) | 5,168 (12.0%) | 11,930 (12.3%) | 5,425 (9.9%) | 1,613 (10.8%) | 10,183 (8.8%) |
| Hypertension, N (%) | 20,667 (47.9%) | 48,403 (49.8%) | 23,814 (43.6%) | 6,890 (46.1%) | 47,167 (40.8%) |
| Type 2 diabetes, N (%) | 9,521 (22.1%) | 23,542 (24.2%) | 11,976 (21.9%) | 3,200 (21.4%) | 22,046 (19.1%) |
| BMI, mean (SD) | 27.7 (7.3) | 29.0 (7.2) | 29.4 (7.2) | 29.6 (7.0) | 29.6 (7.1) |
| BMI, median (IQR) | 27.2 (22.9 - 32.0) | 28.28 (24.0 - 33.2) | 28.79 (24.6 - 33.8) | 28.89(24.9 - 33.9) | 28.99 (24.9 - 33.9) |
| Smoking status, N (%) | | | | | |
| Current | 1,517 (3.5%) | 3,599 (3.7%) | 2,301 (4.2%) | 644 (4.3%) | 4,378 (3.8%) |
| Former | 1,403 (3.3%) | 3,124 (3.2%) | 1,508 (2.8%) | 480 (3.2%) | 2,840 (2.5%) |
| Never | 1,894 (4.4%) | 3,820 (3.9%) | 2,095 (3.8%) | 705 (4.7%) | 3,948 (3.4%) |
| Unknown | 38,326 (88.8%) | 86,659 (89.2%) | 48,725 (89.2%) | 13,108 (87.8%) | 104,315 (90.3%) |
| **Healthcare Resource Utilization in Baseline** | | | | | |
| Patients with OP visit, N (%) | 42,864 (99.4%) | 95,676 (98.4%) | 52,986 (97.0%) | 14,729 (98.6%) | 110,460 (95.7%) |
| Number of all-cause OP visits, mean (SD) | 8.1 (9.2) | 8.4 (10.0) | 7.7 (9.7) | 8.6 (10.7) | 6.9 (8.9) |
| Patients with ED visit, N (%) | 7,516 (17.4%) | 19,768 (20.3%) | 11,673 (21.4%) | 3,062 (20.5%) | 22,014 (19.1%) |
| Number of all-cause ED visits, mean (SD) | 0.51 (4.82) | 0.55 (3.57) | 0.54 (3.00) | 0.52 (3.58) | 0.46 (2.92) |

| | | | | | |
|---|---|---|---|---|---|
| Patients with IP admission, N (%) | 3,986 (9.2%) | 10,772 (11.1%) | 5,855 (10.7%) | 1,697 (11.4%) | 10,904 (9.4%) |
| Number of all-cause IP admissions, mean (SD) | 0.16 (0.86) | 0.20 (0.92) | 0.21 (1.10) | 0.22 (1.03) | 0.19 (1.09) |

[1]Age as of 2020
BMI: body mass index; CCI: Charlson Comorbidity Index; ED: emergency department; IP: inpatient; IQR: interquartile range; OP: outpatient; SD: standard deviation

**Table S9. Demographic and clinical characteristics of individuals continuously enrolled in the Linked Dataset according to vaccination status in 2020-2021**

| Variable | Vaccinated 2020 - 2021 | Unvaccinated 2020 - 2021 |
|---|---|---|
| | N=138,824 | N=186,565 |
| Age in y,[1] mean, SD | 55.0 (22.9) | 54.3 (20.5) |
| Age in y,[1] median (IQR) | 61 (45 - 72) | 58 (44 - 69) |
| Age group, N (%) | | |
| 5 – 12 y | 12,797 (9.2%) | 9,480 (5.1%) |
| 13 – 17 y | 6,771 (4.9%) | 6,884 (3.7%) |
| 18 – 49 y | 21,162 (15.2%) | 45,359 (24.3%) |
| 50 – 64 y | 43,977 (31.7%) | 63,782 (34.2%) |
| 60 – 64 y | 18,961 (13.7%) | 23,367 (12.5%) |
| 65+ y | 54,117 (39.0%) | 61,060 (32.7%) |
| 65 – 69 y | 13,075 (9.4%) | 15,971 (8.6%) |
| 65 – 74 y | 27,511 (19.8%) | 31,910 (17.1%) |
| 75 – 84 y | 19,418 (14.0%) | 20,681 (11.1%) |
| 85+ y | 7,188 (5.2%) | 8,469 (4.5%) |
| Sex (N, %) | | |
| Female | 79,433 (57.2%) | 109,072 (58.5%) |
| Male | 59,391 (42.8%) | 77,493 (41.5%) |
| Race (N, %) | | |

| | | |
|---|---|---|
| White | 97,085 (69.9%) | 120,835 (64.8%) |
| Asian | 5,533 (4.0%) | 4,874 (2.6%) |
| Black | 9,776 (7.0%) | 19,801 (10.6%) |
| Other | 7,050 (5.1%) | 10,346 (5.5%) |
| Unknown/not reported | 19,380 (14.0%) | 30,709 (16.5%) |
| Ethnicity (N, %) | | |
| Hispanic | 12,725 (9.2%) | 18,983 (10.2%) |
| Non-Hispanic | 118,038 (85.0%) | 159,275 (85.4%) |
| Unknown/not reported | 8,061 (5.8%) | 8,307 (4.5%) |
| Baseline health status | | |
| High-risk patients (N, %) | 94,227 (67.9%) | 117,990 (63.2%) |
| Non-high-risk patients (N, %) | 44,597 (32.1%) | 68,575 (36.8%) |
| CCI (Mean, SD) | 1.20 (1.77) | 1.09 (1.71) |
| CCI conditions (N, %) | | |
| Myocardial infarction | 3,558 (2.6%) | 4,294 (2.3%) |
| Congestive heart failure | 9,064 (6.5%) | 11,391 (6.1%) |
| Peripheral vascular disease | 13,972 (10.1%) | 16,564 (8.9%) |
| Cerebrovascular disease | 8,619 (6.2%) | 10,431 (5.6%) |
| Dementia | 2,821 (2.0%) | 4,207 (2.3%) |
| Chronic pulmonary disease | 24,325 (17.5%) | 29,615 (15.9%) |
| Rheumatic disease | 5,240 (3.8%) | 6,716 (3.6%) |
| Peptic ulcer disease | 1,214 (0.9%) | 1,471 (0.8%) |

| | | |
|---|---|---|
| Mild liver disease | 6,974 (5.0%) | 8,518 (4.6%) |
| Diabetes without chronic complications | 30,952 (22.3%) | 38,369 (20.6%) |
| Renal disease, mild to moderate | 10,329 (7.4%) | 11,978 (6.4%) |
| Diabetes with chronic complications | 14,576 (10.5%) | 16,407 (8.8%) |
| Hemiplegia or paraplegia | 1,126 (0.8%) | 1,687 (0.9%) |
| Any malignancy | 10,280 (7.4%) | 11,741 (6.3%) |
| Moderate to severe liver disease | 402 (0.3%) | 530 (0.3%) |
| Severe renal disease | 1,615 (1.2%) | 2,169 (1.2%) |
| HIV infection, no AIDS | 563 (0.4%) | 768 (0.4%) |
| Metastatic solid tumor | 1,116 (0.8%) | 1,469 (0.8%) |
| AIDS | 67 (0.0%) | 84 (0.0%) |
| Baseline cardiovascular and other risk | | |
| Asthma and chronic obstructive pulmonary disease, N (%) | 21,939 (15.8%) | 26,426 (14.2%) |
| Myocardial infarction–related IP admission, N (%) | 679 (0.5%) | 913 (0.5%) |
| Ischemic stroke–related IP admission, N (%) | 506 (0.4%) | 775 (0.4%) |
| Transient ischemic attack–related IP admission, N (%) | 205 (0.1%) | 287 (0.2%) |
| Hypercholesteremia, N (%) | 16,658 (12.0%) | 17,661 (9.5%) |
| Hypertension, N (%) | 66,348 (47.8%) | 80,593 (43.2%) |
| Type 2 diabetes, N (%) | 31,411 (22.6%) | 38,874 (20.8%) |
| BMI, mean (SD) | 28.8 (7.2) | 29.5 (7.2) |
| BMI, median (IQR) | 28 (24.0 - 33.0) | 28.74 (24.6 - 33.8) |

| | | |
|---|---|---|
| Smoking status, N (%) | | |
| Current | 4,763 (3.4%) | 7,676 (4.1%) |
| Former | 4,238 (3.1%) | 5,117 (2.7%) |
| Never | 5,732 (4.1%) | 6,730 (3.6%) |
| Unknown | 124,091 (89.4%) | 167,042 (89.5%) |
| Healthcare Resource Utilization in Baseline | | |
| Patients with OP visit, N (%) | 136,848 (98.6%) | 179,867 (96.4%) |
| Number of all-cause OP visits, mean (SD) | 8.3 (10.0) | 7.3 (9.1) |
| Patients with ED visit, N (%) | 25,575 (18.4%) | 38,458 (20.6%) |
| Number of all-cause ED visits, mean (SD) | 0.49 (3.8) | 0.52 (3.2) |
| Patients with IP admission, N (%) | 13,608 (9.8%) | 19,606 (10.5%) |
| Number of all-cause IP admissions, mean (SD) | 0.17 (0.87) | 0.21 (1.10) |

[1]Age as of 2020.

BMI: body mass index; CCI: Charlson Comorbidity Index; ED: emergency department; IP: inpatient; IQR: interquartile range; OP: outpatient; SD: standard deviation

**Table S10. Differences in patient characteristics by 2020-2021 influenza vaccination status and vaccination history**

| Variable | Vaccinated 2020 - 2021 | Unvaccinated 2020-2021 | SMD |
|---|---|---|---|
| **Consistently vaccinated** | | | |
| Age[1] | | | |
| Age, mean (SD) | 53.5 (27.0) | 56.7 (25.6) | 0.118 |
| 5 - 12 y, N (%) | 5,540 (15.3%) | 723 (10.3%) | 0.151 |
| 18 – 49 y, N (%) | 3,350 (9.3%) | 878 (12.4%) | 0.102 |
| State of residence | | | |
| Pennsylvania, N (%) | 6,578 (18.2%) | 922 (13.1%) | 0.140 |
| Tennessee, N (%) | 1,392 (3.9%) | 588 (8.4%) | 0.190 |
| Baseline health status | | | |
| CCI, mean (SD) | 1.2 (1.7) | 1.4 (2.0) | 0.109 |
| Peripheral vascular disease, N (%) | 3,993 (11.1%) | 848 (12.1%) | 0.109 |
| Dementia, N (%) | 707 (2.0%) | 266 (3.8%) | 0.110 |
| Healthcare utilization | | | |
| Patients with ED visit, N (%) | 6,048 (16.7%) | 1,468 (20.9%) | 0.107 |
| **Previous adopters** | | | |
| Age[1] | | | |
| 18 – 49 y, N (%) | 8,363 (14.3%) | 6,969 (18.1%) | 0.104 |

| | | | |
|---|---|---|---|
| **Inconsistently vaccinated** | | | |
| Age[1] | | | |
| 18 - 49 y, N (%) | 4,153 (20.7%) | 8,751 (25.3%) | 0.110 |
| 50 – 64 y, N (%) | 7,459 (37.2%) | 10,989 (31.8%) | 0.113 |
| State of Residence | | | |
| California, N (%) | 3,415 (17.0%) | 4,506 (13.0%) | 0.111 |
| **Vaccinated 2019 - 2020 only** | | | |
| Age[1] | | | |
| Age, y, mean (SD) | 56.3 (18.5) | 53.2 (19.8) | 0.164 |
| 18 – 49 y, N (%) | 1,553 (20.4%) | 2,018 (27.5%) | 0.166 |
| 65+ y, N (%) | 2,603 (34.2%) | 2,159 (29.4%) | 0.104 |
| **Not vaccinated** | | | |
| State of residence | | | |
| California, N (%) | 2,812 (17.2%) | 12,982 (13.1%) | 0.114 |

[1]Age as of 2020.

CCI: Charlson Comorbidity Index; ED: emergency department; SD: standard deviation; SMD: standard mean difference

**Figure S1. Age distribution of the included population during**

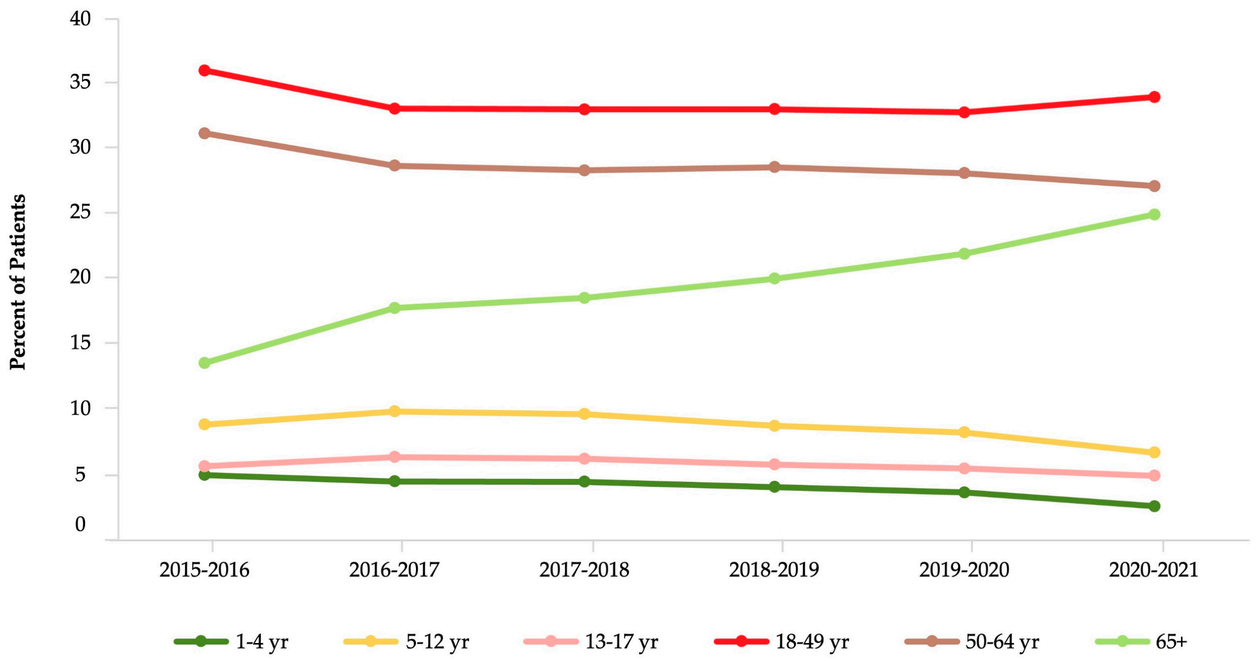

**Figure S2. Timing of influenza vaccinations (A) August 1 through January 31 and (B) before October 31 vs. after October 31, 2015-2016 through 2020-2021 influenza seasons**

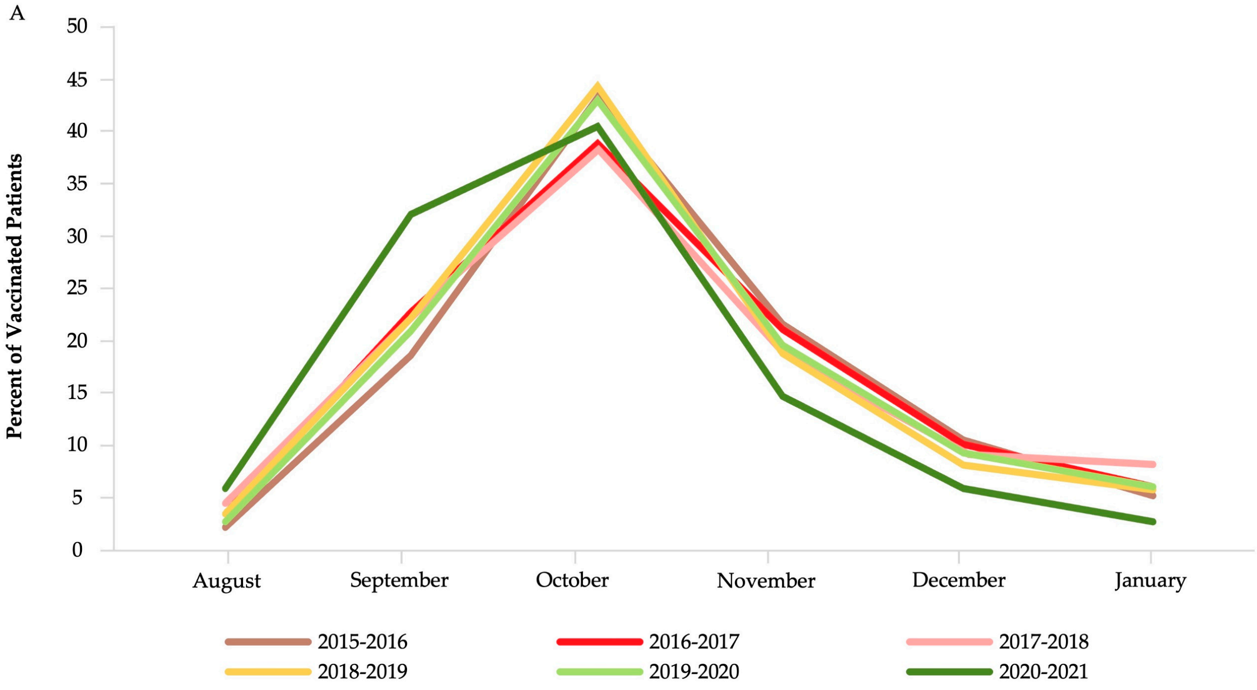

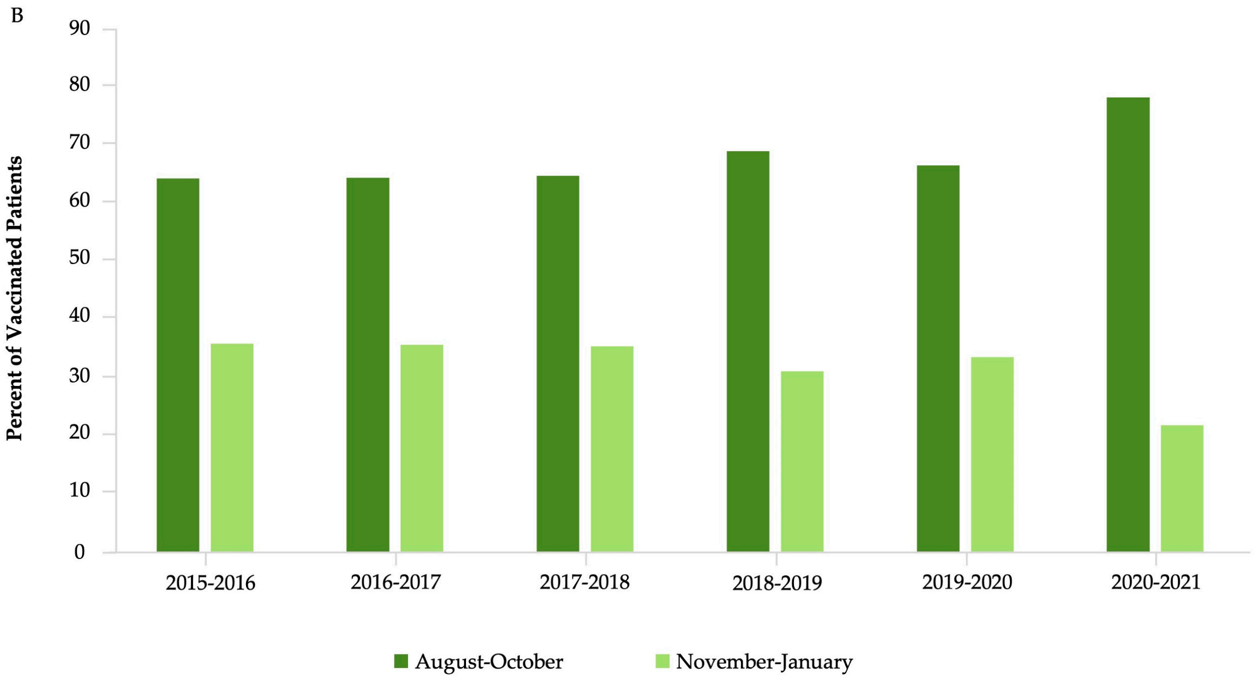
B
Percent of Vaccinated Patients
0
10
20
30
40
50
60
70
80
90
2015-2016
2016-2017
2017-2018
2018-2019
2019-2020
2020-2021
August-October
November-January

**Figure S3. Change in influenza vaccination uptake between 2019-2020 and 2020-2021**

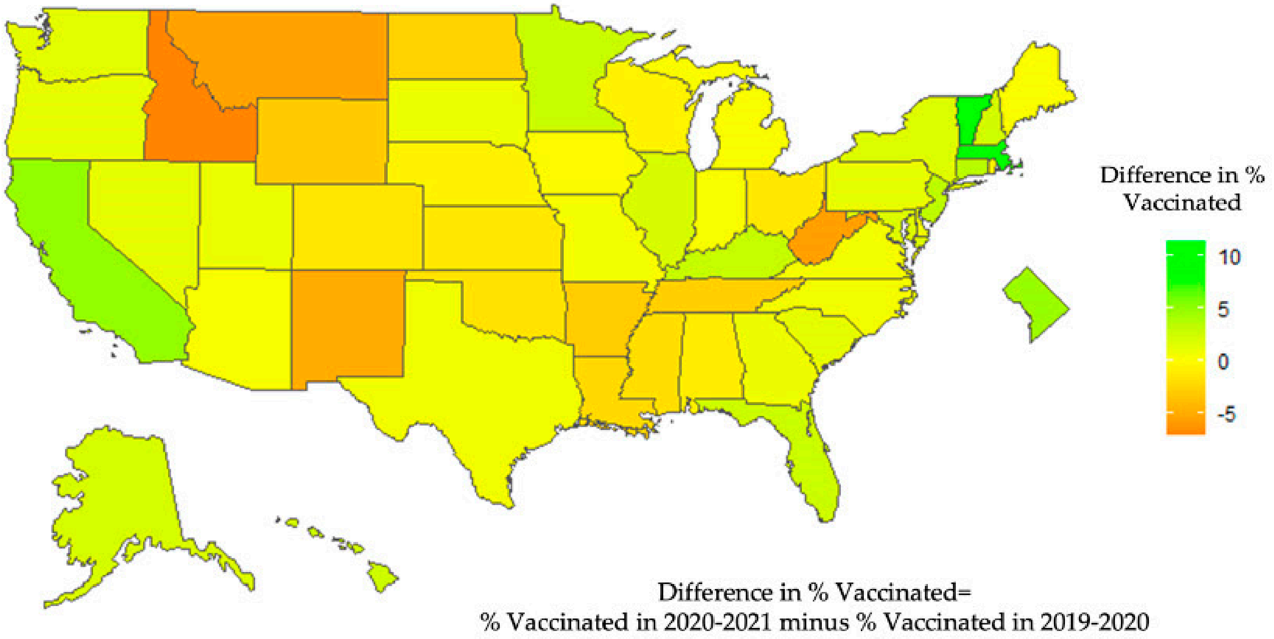

**References**


1. Centers for Disease Control and Prevention. People at higher risk of flu complications. Available online: https://www.cdc.gov/flu/highrisk/index.htm (accessed on 5 February 2022).
